# Sacrococcygeal Pilonidal Sinus Disease: A Decade‐In‐Review of Patient‐Reported Outcome Measures

**DOI:** 10.1111/ans.70330

**Published:** 2025-10-11

**Authors:** Munyaradzi G. Nyandoro, Mary M. K. Teoh, Ellen G. Maclean, Andrew Thompson, David Fletcher

**Affiliations:** ^1^ The University of Western Australia Perth Australia; ^2^ Fiona Stanley Hospital Perth Australia; ^3^ Sir Charles Gairdner Hospital Perth Australia; ^4^ Rockingham General Hospital Perth Australia; ^5^ Harry Perkins Institute of Medical Research Fiona Stanley Hospital Perth Australia

## Abstract

**Background:**

Sacrococcygeal pilonidal sinus disease (SPD) is a chronic inflammatory condition that can significantly impact quality of life. While recurrence and surgical site infection (SSI) rates are well documented, less is known about how different surgical techniques affect patient‐reported outcome measures (PROMs).

**Methods:**

This study employed mixed methods, including standardised mail questionnaires and telephone interviews (May–September 2020), to conduct a multi‐centre retrospective decade follow‐up review of definitive SPD surgery participants from Western Australia. Six surgical technique groups were analysed: Karydakis flap (KF), modified Karydakis flap (MKF), Limberg flap (LF), modified Limberg flap (MLF), other flap techniques (OFT) and secondary intention healing techniques (SIT). PROMs assessed included body image, cosmesis, confidence, functional recovery and overall satisfaction—secondary outcomes examined recurrence, SSI and other complications.

**Results:**

A total of 136 patients met the inclusion criteria. MLF and OFT achieved the fastest functional recovery, with median return‐to‐work times of 14 and 21 days, respectively, compared to 60 days for SIT (*p* = 0.007). MKF and LF achieved the highest body image and cosmetic satisfaction scores (medians of 20 and 19), whereas SIT and OFT scored the lowest. MKF yielded the highest overall satisfaction (100%). SIT was associated with the highest recurrence rate (52.2%) and SSI rates (30.4%), while MLF and MKF combined had low recurrence rates with favourable PROMs.

**Conclusion:**

Flap‐based techniques, particularly the MKF and MLF, offer an optimal balance of functional recovery, cosmetic outcomes and low recurrence rates. Incorporating PROMs into SPD surgical planning can better align treatment with patient priorities and the underlying pathophysiology of the disease.

## 1 Introduction

Sacrococcygeal pilonidal sinus disease (SPD) is a chronic inflammatory condition of the natal cleft characterised by sinus tract formation, most often affecting young adults in their second and third decades of life [1, 2, 3, 4, 5]. Globally, the annual incidence is estimated to be 27–30 cases per 100 000 population, with a notable male predominance [1, 2, 3, 6, 7]. SPD is associated with pain, discharge, recurrent infection and a significant psychosocial burden, particularly impacting body image and self‐esteem [6, 8].

Traditional teaching has cited risk factors such as obesity, hair density and hygiene; however, these associations are inconsistently supported by evidence [6, 9]. Increasingly, basic scientific and clinical data support a mechanical model in which loose hairs become trapped within a substantial contact surface of the intergluteal fold [1, 2, 5, 6, 9]. This configuration, rather than just ‘depth’, encourages hair entrapment and both factors appear to be necessary for disease development [10, 11]. Recognising this mechanism can guide surgical planning and consent discussions. When minimally invasive techniques fail, flap procedures that decrease the contact surface area may target the underlying risk, even if that is not their primary goal.

A wide range of surgical procedures is used for SPD, from minimally invasive pit excisions to complex flap reconstructions [7, 8, 12, 13, 14, 15, 16, 17, 18]. International guidelines recommend a ‘step‐up’ strategy—reserving more complex flap procedures for recurrent or complicated disease—yet in some Australian centres, flaps remain a standard first‐line treatment even for primary presentations [12, 15, 19, 20, 21, 22].

Historically, outcomes have been reported in terms of recurrence, infection and wound healing, which may not accurately reflect the patient's perspective on surgical success [16, 23, 24, 25]. Patient‐reported outcomes (PROMs)—such as body image, cosmetic satisfaction, confidence and functional recovery—are increasingly recognised as essential in evaluating interventions.

To the author's knowledge, no study in Australia has compared PROMs across a spectrum of different surgical techniques for the elective definitive management of SPD. Procedure choice often depends on surgeon preference, with limited evidence to guide patient‐centred decision‐making. This study aimed to compare PROMs and secondary clinical outcomes for six common elective SPD techniques in Western Australia: Karydakis flap (KF), Modified Karydakis flap (MKF), Limberg flap (LF), Modified Limberg flap (MLF), other flap techniques (OFT) and secondary intention techniques (SIT). We hypothesised that the surgical technique would influence PROMs as well as recurrence and complication rates, with potential implications for aligning local practice with guideline‐based pathways.

## 2 Methods

### 2.1 Study Design and Setting

This study conducted a mixed methods mail and telephone questionnaire between May and September 2020 on a multi‐centre retrospective cohort of participants who underwent elective definitive SPD surgery between 2010 and 2019 in three Western Australian metropolitan health regions. Each centre used a range of standardised operative techniques, with variations depending on surgeon preference and patient presentation [26].

### 2.2 Participants

Seven hundred seventy‐four patients who underwent SPD surgery between January 2010 and December 2019 were identified. Patients were grouped according to procedure type: Karydakis flap (KF, *n* = 302), Modified Karydakis (MKF, *n* = 242), Limberg flap (LF, *n* = 52), Modified Limberg (MLF, *n* = 40), other flap techniques (OFT, *n* = 48) and secondary intention techniques (SIT, *n* = 90).

Eligible patients were identified from theatre records using relevant procedural codes. Inclusion criteria were: (1) age of 16 years or older; (2) elective surgery for SPD performed with one of six predefined techniques—KF, MKF, LF, MLF, OFT, SIT; and (3) at least 12 months of follow‐up. Exclusion criteria included acute abscess drainage without definitive sinus surgery, incomplete operative records, or an inability to contact the patient for PRO assessment.

### 2.3 Sample Size and Data Collection

A priori sample size calculation indicated that 20 participants per procedure group were necessary to detect significant Spearman correlations between covariates with *α* = 0.05 and *β* = 0.2. A quasi‐randomised sample of 50 participants was generated through a computerised random cohort enquiry (accounting for a 40% response rate) from each group, including all available cases for LF, MLF and OFT, leading to a total of 300 study participants. PROMs were collected via a combination of hard‐copy mail questionnaires and structured telephone interviews.

**FIGURE 1:**
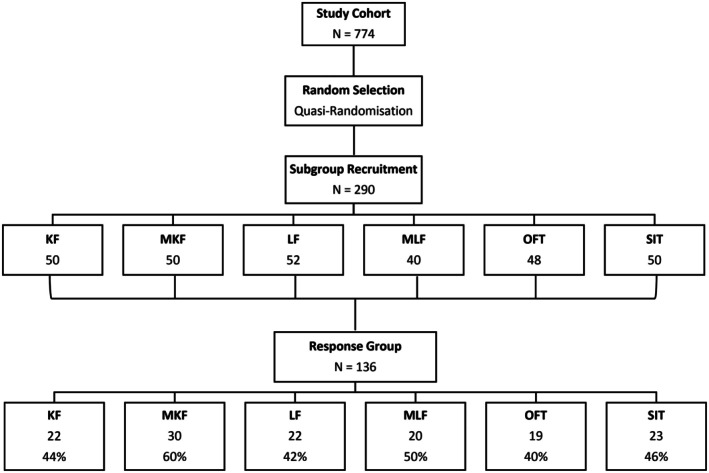
Participant recruitment and follow‐up.

Three hundred invitation packages—containing participant information sheets, questionnaires, consent forms and reply‐paid return envelopes addressed to the research team—were posted. A log was maintained of all forms not yet received. Structured follow‐up telephone interviews, conducted by trained research staff blinded to surgical technique, aimed to improve overall study participation and response rates. These standardised interviews assessed body image, cosmetic satisfaction, return to work, overall satisfaction and confidence before and after surgery (Table S1). Of these, 136 responses (45.3%) were received, meeting the minimum of 20 participants per group (Figure 1).

All participants provided consent, either explicitly by taking part in the telephone interview or implicitly by returning a completed questionnaire. Hospital records were reviewed to extract demographic data, procedure type, operative time, length of stay, use of drains, administration of antibiotics and postoperative complications. Recurrence was defined according to Doll's criteria (≥ 1 hard sign or ≥ 2 soft signs post‐healing) [27]. All epidemiological data were recorded at the time of admission for primary SPD surgery.

### 2.4 Outcomes

The key patient‐focused outcome measures included: (i) aesthetic perceptions of body image (BIS), (ii) cosmesis (CS) and quality of life (QoL) issues, (iii) time to painless mobilisation (PM), (iv) painless sit on toilet (SoT), (v) cessation of analgesia (CoA), (vi) return to usual activities (RuA) and (vii) return to work (RtW) and these were correlated with patient satisfaction and confidence as expressed in the long‐term follow‐up. These outcome measures were scored using published and validated tools for SPD procedures. The study also assessed patients' perceptions of the surgical informed consent process using a Likert scale. Patients were invited to provide additional comments about their surgical experiences, and this qualitative data was analysed thematically for common themes.

### 2.5 Surgical Technique Definitions

Minimally invasive procedures were not carried out at the participating centres during the study period, and most cases used flap/excision techniques in accordance with local practice patterns. Full operative definitions for all surgical techniques, including incision patterns, tissue handling, closure methods and postoperative care, are detailed in Table S2.

### 2.6 Statistical Analysis

Baseline characteristics and outcome data for each procedure group were described using means (±standard deviations), medians (interquartile ranges), or frequencies/proportions (%), depending on the distribution. Continuous variables were compared using Kruskal–Wallis tests (with Benjamini–Hochberg adjusted *p*‐values) and Mann–Whitney *U* tests. Categorical variables were analysed with *χ* ^2^ or Fisher's exact tests. Spearman's rank correlation test was employed to assess the correlation between two quantitative variables. Significance was set at *p* < 0.05 (two‐tailed). Analyses were performed using SPSS Statistics (IBM Corp., Armonk, N.Y., USA).

### 2.7 Ethics

Ethics approval was granted by the lead Human Research Ethics Committee (HREC)—South Metropolitan Health Service Ethics (SMHS)—RGS511 and The University of Western Australia Human Research Ethics Committee RA/4/20/4547. Relevant site‐specific governance clearance for access to each hospital site was also granted.

## 3 Results

### 3.1 Demographics

Of 136 respondents (77.9% male), most were non‐diabetic (90%), non‐hirsute (67%) and had no compliance issues (Table 1). A third were obese (33.8%) and over half were current smokers (53.7%). The median age was 25.5 years (IQR 16–50), with a BMI of 27.6 kg/m^2^ (IQR 19–50) (Table 2).

**TABLE 1:** Participant demographics per SPD surgery types.

*N* = 136			Total sample	KF	MKF	LF	MLF	OFT	SIT	*p*
	Total sample	*N* =	136	22	30	22	20	19	23	
Gender	Male	*n* =	106	16	22	18	16	16	18	0.925
		%	77.9%	72.7%	73.3%	81.8%	80.0%	84.2%	78.3%	
	Female	*n* =	30	6	8	4	4	3	5	
		%	22.1%	27.3%	26.7%	18.2%	20.0%	15.8%	21.7%	
BMI group	Healthy weight	*n* =	40	7	8	7	5	8	5	0.771
		%	29.4%	31.8%	26.7%	31.8%	25.0%	42.1%	21.7%	
	Overweight	*n* =	50	8	14	6	8	7	7	
		%	36.8%	36.4%	46.7%	27.3%	40.0%	36.8%	30.4%	
	Obese	*n* =	46	7	8	9	7	4	11	
		%	33.8%	31.8%	26.7%	40.9%	35.0%	21.1%	47.8%	
Age group	15–19 years	*n* =	20	4	6	3	2	3	2	0.931
		%	14.7%	18.2%	20.0%	13.6%	10.0%	15.8%	8.7%	
	20–29 years	*n* =	70	10	13	11	12	12	12	
		%	51.5%	45.5%	43.3%	50.0%	60.0%	63.2%	52.2%	
	30+ years	*n* =	46	8	11	8	6	4	9	
		%	33.8%	36.4%	36.7%	36.4%	30.0%	21.1%	39.1%	
Employed or/School	No	*n* =	13	2	2	5	1	1	2	0.346
		%	9.6%	9.1%	6.7%	22.7%	5.0%	5.3%	8.7%	
	Yes	*n* =	123	20	28	17	19	18	21	
		%	90.4%	90.9%	93.3%	77.3%	95.0%	94.7%	91.3%	
Diabetes	No	*n* =	122	20	29	21	20	13	19	0.009*
		%	89.7%	90.9%	96.7%	95.5%	100%	68.4%	82.6%	
	Yes	*n* =	14	2	1	1	0	6	4	
		%	10.3%	9.1%	3.3%	4.5%	0.0%	31.6%	17.4%	
Hirsutism	No	*n* =	91	10	23	19	16	7	16	0.002*
		%	66.9%	45.5%	76.7%	86.4%	80.0%	36.8%	69.6%	
	Yes	*n* =	45	12	7	3	4	12	7	
		%	33.1%	54.5%	23.3%	13.6%	20.0%	63.2%	30.4%	
Smoker	No	*n* =	63	11	17	8	9	8	10	0.775
		%	46.3%	50.0%	56.7%	36.4%	45.0%	42.1%	43.5%	
	Yes	*n* =	73	11	13	14	11	11	13	
		%	53.7%	50.0%	43.3%	63.6%	55.0%	57.9%	56.5%	
Ex‐smoker recent < 3 months	No	*n* =	115	20	24	19	15	18	19	0.523
		%	84.6%	90.9%	80.0%	86.4%	75.0%	94.7%	82.6%	
	Yes	*n* =	21	2	6	3	5	1	4	
		%	15.4%	9.1%	20.0%	13.6%	25.0%	5.3%	17.4%	
Mental health/compliance	No	*n* =	125	19	30	18	19	19	20	0.099
		%	91.9%	86.4%	100%	81.8%	95.0%	100%	87.0%	
	Yes	*n* =	11	3	0	4	1	0	3	
		%	8.1%	13.6%	0.0%	18.2%	5.0%	0.0%	13.0%	
Region	SMHS	*n* =	56	9	16	9	4	5	11	0.019*
		%	41.2%	40.9%	53.3%	40.9%	20.0%	26.3%	56.6%	
	EMHS	*n* =	29	8	6	3	6	1	5	
		%	21.3%	36.4%	20.0%	13.6%	30.0%	5.3%	21.7%	
	NMHS	*n* =	51	5	8	10	10	13	5	
		%	37.5%	22.7%	26.7%	45.5%	50.0%	68.4%	21.7%	

*Note*: Values are number of patients (%) unless otherwise indicated. Pearson Chi‐square analysis and Fisher's exact test (for cell values < 5) (*Bolded denotes significance at *p* < 0.05).

Abbreviations: BMI, body mass index; SMHS, EMHS, NMHS, South, East, North Metropolitan Health Services; SPD, sacrococcygeal pilonidal sinus disease.

**TABLE 2:** Initial surgery details per SPD surgery type—demographic continuous variables.

*N* = 136		Total sample	KF	MKF	LF	MLF	OFT	SIT	*p*
	*N* =	136	22	30	22	20	19	23	
Initial length of stay (days)	*M*	1.5	0.8	0.9	1.4	1.5	3.7	1.0	< **0.001**
	SD	1.7	1.0	0.6	1.7	1.2	2.8	0.7	
	Mdn	1.0	1.0	1.0	1.0	1.0	3.0	1.0	
	IQR	0–9	0–3	0–2	0–6	0–6	0–9	0–3	
Procedure duration (min)	*M*	47.4	49.4	44.6	43.2	44.8	75.2	32.6	**0.002**
	SD	28.2	21.6	16.3	9.9	20.8	54.1	16.9	
	Mdn	43.5	49.5	45.0	41.5	42.0	62.0	30.0	
	IQR	10–250	20–90	20–90	27–62	10–100	20–250	10–65	
BMI	*M*	28.4	28.9	27.6	28.3	28.7	27.2	30.2	0.645
	SD	6.0	6.8	4.9	4.9	6.0	7.0	6.6	
	Mdn	27.6	26.6	27.6	29.5	27.0	27.0	29.0	
	IQR	19–50	20–46	19–40	21–38	19–46	20–49	23–50	
Age	*M*	27.0	27.5	25.3	28.7	27.6	24.9	28.5	0.363
	SD	7.5	6.9	7.3	8.5	7.3	6.5	8.2	
	Mdn	25.5	28.0	24.0	27.0	26.0	23.0	25.0	
	IQR	16–50	16–44	16–47	16–44	17–45	17–45	16–50	
Phone follow‐up (years)	*M*	3.4	2.9	2.7	3.5	3.5	4.3	3.8	0.501
	SD	1.3	0.6	0.5	0.9	0.9	2.4	1.0	
	Mdn	3.0	3.0	3.0	3.0	3.0	3.0	4.0	
	IQR	2–10	2–4	2–3	2–5	2–5	2–10	2–6	
Clinic follow‐up (weeks)	*M*	8.4	7.8	5.4	9.3	5.2	13	11	**0.001**
	SD	10.6	5.9	5.3	14.8	4.3	16.8	10	
	Mdn	6.0	6.5	3.0	6.0	3.0	7.0	8.0	
	IQR	1–72	2–22	1–22	2–72	1–16	2–63	3–52	
Drain^a^ duration (days)	*M*	3.0	2.3	2.1	2.2	2.5	5.6	—	< **0.001**
	SD	3.1	1.4	1.6	1.8	1.3	5.0	—	
	Mdn	2.0	3.0	2.0	1.0	2.0	4.5	—	
	IQR	1–21	1–5	1–7	1–6	1–9	1–21	—	
HITH length of stay (days)	*M*	21.0	13.1	12.4	18.7	16.0	15.1	32.5	< **0.001**
	SD	18.3	8.3	8.6	16.0	5.0	5.2	25.2	
	Mdn	14.0	14.0	8.5	14.0	14.0	14.0	21.0	
	IQR	4–80	5–30	7–30	7–60	9–21	7–21	4–80	
GP^b^ length of stay (days)	*M*	12.9	13.5	—	14.0	12.7	10.5	—	< **0.001**
	SD	2.2	1.0	—	0	2.3	5.0	—	
	Mdn	14.0	14.0	—	14.0	14.0	10.5	—	
	IQR	7–14	12–14	—	14–14	10–14	7–14	—	

*Note*: Independent‐samples Kruskal–Wallis's test with Benjamini–Hochberg adjusted *p*‐values (*Bolded denotes significance at *p* < 0.05).

Abbreviations: BMI, body mass index; HITH, hospital‐in‐the‐home; IQR, interquartile range; *M*, mean; Mdn, median; nn, number; SD, standard deviation; SPD, sacrococcygeal pilonidal sinus disease.

^a^ SIT did not have a drain in place.

^b^ MKF and SIT were not sent to GP for follow‐up.

### 3.2 Operative Characteristics

Operative time varied significantly between techniques (*p* < 0.001, Table 2). SIT had the shortest median operative time at 30 min (IQR 10–65), whereas OFT had the longest at 62 min (IQR 20–250). Drains were not used in SIT and remained longest in OFT (4.5 days, IQR 1–21) and shortest in LF (1 day, IQR 1–6) (*p* < 0.001). The median length of stay was longest for OFT at 3 days (IQR 0–9) and 1 day for all other groups (*p* < 0.001) (Table 2).

### 3.3 Postoperative Complications and Recurrence

The overall SSI rate was 40.4% (*n* = 55), with no significant difference between procedures (*p* = 0.117, Table 3). Wound dehiscence occurred in 32.4% (*n* = 44) of patients, most commonly in OFT (52.6%) and least in MLF (25%) (*p* = 0.002). Recurrence rates, defined according to Doll's criteria, varied significantly (*p* < 0.001), with OFT having the highest recurrence at 63.2% and MLF the lowest at 5.0%.

**TABLE 3:** Summarised postoperative complications and outcomes per SPD surgery type.

*N* = 136			Total sample	KF	MKF	LF	MLF	OFT	SIT	*p*
	Total sample	*N* =	136	22	30	22	20	19	23	
Recurrence (any time)	Yes	*n* =	35	5	3	4	1	12	10	< 0.001*
		%	25.7%	22.7%	10.0%	18.2%	5.0%	63.2%	43.5%	
Recurrence (< 3 months)	Yes	*n* =	18	3	3	2	0	6	4	0.088
		%	51.4%	60.0%	100%	50.0%	0.0%	50.0%	40.0%	
Recurrence (4–6 months)	Yes	*n* =	8	2	0	0	0	3	3	
		%	22.9%	40.0%	0.0%	0.0%	0.0%	25.0%	30.0%	
Recurrence (7–12 months)	Yes	*n* =	4	0	0	2	1	1	0	
		%	11.4%	0.0%	0.0%	50.0%	100%	8.3%	0.0%	
Recurrence (> 12 months)	Yes	*n* =	5	0	0	0	0	2	3	
		%	14.3%	0.0%	0.0%	0.0%	0.0%	16.7%	30.0%	
Surgical site infection	No	*n* =	81	13	20	13	15	6	14	0.117
		%	59.6%	59.1%	66.7%	59.1%	75.0%	31.6%	60.9%	
	Yes	*n* =	55	9	10	9	5	13	9	
		%	40.4%	40.9%	33.3%	40.9%	25.0%	68.4%	39.1%	
Wound dehiscence	No	*n* =	92	12	21	12	15	9	23	0.002*
		%	67.6%	54.5%	70.0%	54.5%	75.0%	47.4%	100%	
	Yes	*n* =	44	10	9	10	5	10	—	
		%	32.4%	45.5%	30.0%	45.5%	25.0%	52.6%	—	
Hematoma	No	*n* =	125	19	29	17	19	18	—	0.059
		%	91.9%	86.4%	96.7%	77.3%	95.0%	94.7%	—	
	Yes	*n* =	11	3	1	5	1	1	0	
		%	8.1%	13.6%	3.3%	22.7%	5.0%	5.3%	0.0%	
Any complication	No	*n* =	61	10	17	10	11	2	11	0.040*
		%	44.9%	45.5%	56.7%	45.5%	55.0%	10.5%	47.8%	
	Yes	*n* =	75	12	13	12	9	17	12	
		%	55.1%	54.5%	43.3%	54.5%	45.0%	89.5%	52.2%	
Complications required surgical intervention (*n* = 75)	No	*n* =	44	8	9	10	7	6	4	0.031*
		%	58.7%	66.7%	69.2%	83.3%	77.8%	35.3%	33.3%	
	Yes	*n* =	31	4	4	2	2	11	8	
		%	41.3%	33.3%	30.8%	16.7%	22.2%	64.7%	66.7%	
Intervention at original hospital (*n* = 31)	No	*n* =	10	0	1	0	2	4	3	0.197
		%	32.3%	0.0%	25.0%	0.0%	100%	36.4%	37.5%	
	Yes	*n* =	21	4	3	2	0	7	5	
		%	67.7%	100%	75.0%	100%	0.0%	63.6%	62.5%	
Overall satisfaction	No	*n* =	5	1	0	1	1	1	1	0.913
		%	3.7%	4.5%	0.0%	4.5%	5.0%	5.3%	4.3%	
	Yes	*n* =	131	21	30	21	19	18	22	
		%	96.3%	95.5%	100%	95.5%	95.0%	94.7%	95.7%	
Recommend to others	No	*n* =	13	1	1	2	3	4	2	0.347
		%	9.6%	4.5%	3.3%	9.1%	15.0%	21.1%	8.7%	
	Yes	*n* =	123	21	29	20	17	15	21	
		%	90.4%	95.5%	96.7%	90.9%	85.0%	78.9%	91.3%	

*Note*: Values are number of patients (%) unless otherwise indicated. Pearson Chi‐square analysis and Fisher's exact test (for cell values < 5) (*Bolded denotes significance at *p* < 0.05).

Abbreviation: SPD, sacrococcygeal pilonidal sinus disease.

### 3.4 Functional Recovery

Patient‐reported functional recovery varied significantly across surgical techniques (Table 4). Median time to painless mobilisation ranged from 7 days in both the LF and MKF groups to 21 days in KF and SIT, with OFT achieving the fastest recovery (median 10 days, IQR 0–14). SIT patients experienced the longest duration to regain painless mobilisation (median 21 days, IQR 0–180), while MKF and LF demonstrated the shortest times (both 7 days, *p* = 0.003).

**TABLE 4:** Quality of life recovery details per SPD surgery type.

*N* = 136		Total sample	KF	MKF	LF	MLF	OFT	SIT	*p*
	*N* =	136	22	30	22	20	19	23	
Painless mobilisation (days)	*M*	19.4	21.3	11.8	12.5	22.3	8.3	40.9	0.003*
	SD	28.3	20.4	14.9	18.6	26.0	4.3	50.8	
	Mdn	10.0	21.0	7.0	7.0	14.0	10.0	21.0	
	IQR	0–180	1–90	1–60	2–90	4–120	0–14	0–180	
Painless sitting (days)	*M*	28.0	28.4	19.9	21.4	30.9	21.3	47.4	0.023*
	SD	31.0	20.4	19.0	25.5	38.1	13.7	49.5	
	Mdn	20.5	24.5	14.0	12.0	21.0	20.0	30.0	
	IQR	3–180	7–90	3–70	7–120	7–180	3–60	7–180	
Analgesia ceased (days)	*M*	18.5	15.1	11.7	9.8	22.5	12.1	40.5	< 0.001*
	SD	31.0	16.0	12.0	12.6	59.7	7.9	38.9	
	Mdn	10.0	10.5	7.0	7.0	7.0	10.0	30.0	
	IQR	1–270	1–60	2–60	1–60	1–270	2–30	4–150	
Return to activities (days)	*M*	36.3	40.6	27.3	29.9	37.0	21.8	61.6	0.013*
	SD	39.7	37.3	24.7	42.4	51.7	12.0	48.7	
	Mdn	21.0	28.0	17.5	14.0	21.0	21.0	49.0	
	IQR	2–240	2–150	5–90	7–210	7–240	3–45	10–180	
Return to work (days)	*M*	40.5	44.1	32.7	27.7	36.3	33.0	69.2	0.007*
	SD	40.4	42.5	27.5	30.3	51.6	23.8	49.7	
	Mdn	28.0	29.0	24.5	14.0	21.0	30.0	60.0	
	IQR	2–240	2–180	4–120	7–150	7–240	10–90	10–180	

*Note*: Kruskal–Wallis's test (with Benjamini–Hochberg adjusted *p*‐values) (*Bolded denotes significance at *p* < 0.05).

Abbreviations: IQR, interquartile range; *M*, mean; Mdn, median; nn, number; SD, standard deviation; SPD, sacrococcygeal pilonidal sinus disease.

Median time to painless sitting was shortest in the LF group (12 days, IQR 7–120) and longest in SIT (30 days, IQR 7–180, *p* = 0.023). MKF also performed well (median 14 days), whereas KF and MLF patients had longer durations (24.5 and 21 days, respectively). Analgesia cessation occurred most rapidly in LF and MLF (both median 7 days), with SIT requiring the longest duration (median 30 days, IQR 4–150, *p* < 0.001).

Return to usual activities was earliest for OFT (median 21 days, IQR 3–45) and MLF (14 days, IQR 7–210) and longest for SIT (49 days, IQR 10–180) (*p* = 0.013). Similarly, return to work was fastest for MLF (median 14 days, IQR 7–150) and slowest for SIT (60 days, IQR 10–180, *p* = 0.007). MKF and LF also achieved a relatively earlier return to work (24.5 and 21 days, respectively) compared with KF (29 days) and OFT (30 days).

### 3.5 Cosmesis, Body Image and Satisfaction

Patient‐reported outcome measures are summarised in Table 5. Median body image scores were highest in KF, MKF and LF (all 20; IQR 14–20, 9–20 and 6–20, respectively) and lowest in OFT (15, IQR 10–20) (*p* = 0.001). Cosmetic satisfaction was greatest in LF (median 19, IQR 4–24) and MKF (median 19, IQR 7–23) and lowest in SIT (median 13, IQR 3–24) (*p* = 0.031). Post‐procedure confidence scores were highest in KF (median 7.5, IQR 1–10) and lowest in OFT (median 5.5, IQR 1–10) (*p* = 0.207). Ranked satisfaction was highest in LF (median 7.5, IQR 1–10) and lowest in SIT (median 5, IQR 1–10) (*p* = 0.032).

**TABLE 5:** Body perception and satisfaction scores per SPD surgery type.

*N* = 136		Total sample	KF	MKF	LF	MLF	OFT	SIT	*p*
	*N* =	136	22	30	22	20	19	23	
Body image score	*M*	17.7	18.4	18.6	18.4	18.1	14.7	17.3	0.001*
	SD	3.4	2.1	3.1	3.3	3.1	3.9	3.7	
	Mdn	19	20	20	20	19	15	19	
	IQR	6–20	14–20	9–20	6–20	6–20	10–20	8–20	
Cosmetic score	*M*	15.9	16.9	17.2	17.4	14.7	13.5	14.9	0.031*
	SD	4.8	4.4	4.3	4.9	5.2	4.7	5.2	
	Mdn	16	17	19	18	15.5	15	13	
	IQR	3–24	4–24	7–23	4–24	3–20	3–20	3–24	
Overall satisfaction	*M*	6.3	6.6	6.8	7.0	5.6	5.4	5.8	0.032*
	SD	2.1	2.0	1.9	2.3	2.0	2.1	2.3	
	Mdn	6	7	7	7.5	6	5.5	5	
	IQR	1–10	1–10	3–10	1–10	1–8	1–9	1–10	
Confidence (before)	*M*	7.0	7.7	7.7	7.2	7.7	4.7	6.5	0.003*
	SD	2.8	2.1	2.6	2.6	2.5	2.2	3.5	
	Mdn	8	8	8	7.5	8	4	8	
	IQR	1–10	1–10	2–10	1–10	1–10	1–8	1–10	
Confidence (after)	*M*	6.2	7.4	6.6	5.7	6.1	5.6	5.7	0.207
	SD	2.7	2.4	2.6	2.5	2.8	2.5	3.0	
	Mdn	6	7.5	6.5	6	6	5.5	6.5	
	IQR	1–10	1–10	2–10	1–10	1–10	1–10	1–10	
SPD Mdn^a^ confidence difference	*p*‐value	0.005*	0.406	0.083	0.068	0.033*	0.061	0.288	

*Note*: Independent‐samples Kruskal–Wallis's test (with Benjamini–Hochberg adjusted *p*‐values) (*Bolded denotes significance at *p* < 0.05).

Abbreviations: IQR, interquartile range; *M*, mean; Mdn, median; nn, number; SD, standard deviation; SPD, sacrococcygeal pilonidal sinus disease.

^a^ Mdn = median related‐samples Wilcoxon signed rank test (*Bolded denotes significance at *p* < 0.05).

Across the cohort, there was a significant overall decline in confidence from before to after surgery (*p* = 0.005). The largest reduction occurred in MLF, where median scores fell from 8 (IQR 2) preoperatively to 6 (IQR 3) postoperatively (*p* = 0.033). No significant pre–post change was observed in KF, LF, MLF, OFT, or SIT.

### 3.6 Surgical Informed Consent

About ¾ of the participants felt fully informed about the procedure/risks/benefits. There was a positive correlation between being fully surgically informed about cosmesis and the likelihood of recommending the SPD procedure to others, overall satisfaction, confidence and postoperative self‐sufficiency in managing their condition (Table 6). However, fewer patients felt adequately informed about recovery (61%) and alternative treatments (46%). Half of the participants strongly agreed that pre‐op clinics thoroughly prepared them for post‐operative self‐care. Only 8% felt they needed more time in pre‐op clinics, with 55% reporting that they now fully understood disease management.

**TABLE 6:** Correlation analysis with SIC—with procedure/risks/benefits and being well informed in clinic as the dependent variables.

*N* = 136	Procedure/risks/benefits		Being well‐informed in clinic	
	Spearman's correlation coefficient rho (*r*_*s*_)	*p*	Spearman's correlation coefficient rho (*r*_*s*_)	*p*
Body image score	0.374	***p*** < **0.001**	0.445	***p*** < **0.001**
Cosmetic score	0.264	***p*** = **0.002**	0.389	***p*** < **0.001**
Recommend to others	0.340	***p*** < **0.001**	0.267	***p*** = **0.002**
Overall satisfaction	0.352	***p*** < **0.001**	0.190	***p*** = **0.026**
Needed more clinic time	−0.394	***p*** < **0.001**	−0.503	***p*** < **0.001**
Post‐op self‐care	0.483	***p*** < **0.001**	0.171	***p*** = **0.046**
Confidence post‐op	0.235	***p*** = **0.006**	0.752	***p*** < **0.001**
Post op complications	0.212	***p*** = **0.013**	−0.203	***p*** = **0.018**

*Note*: Summary of key positive and negative correlation factors. For readability, only significant results were indicate in bold.

Abbreviations: Neg, negative correlations; SIC, surgical informed consent.

### 3.7 Thematic Analysis

**FIGURE 2:**
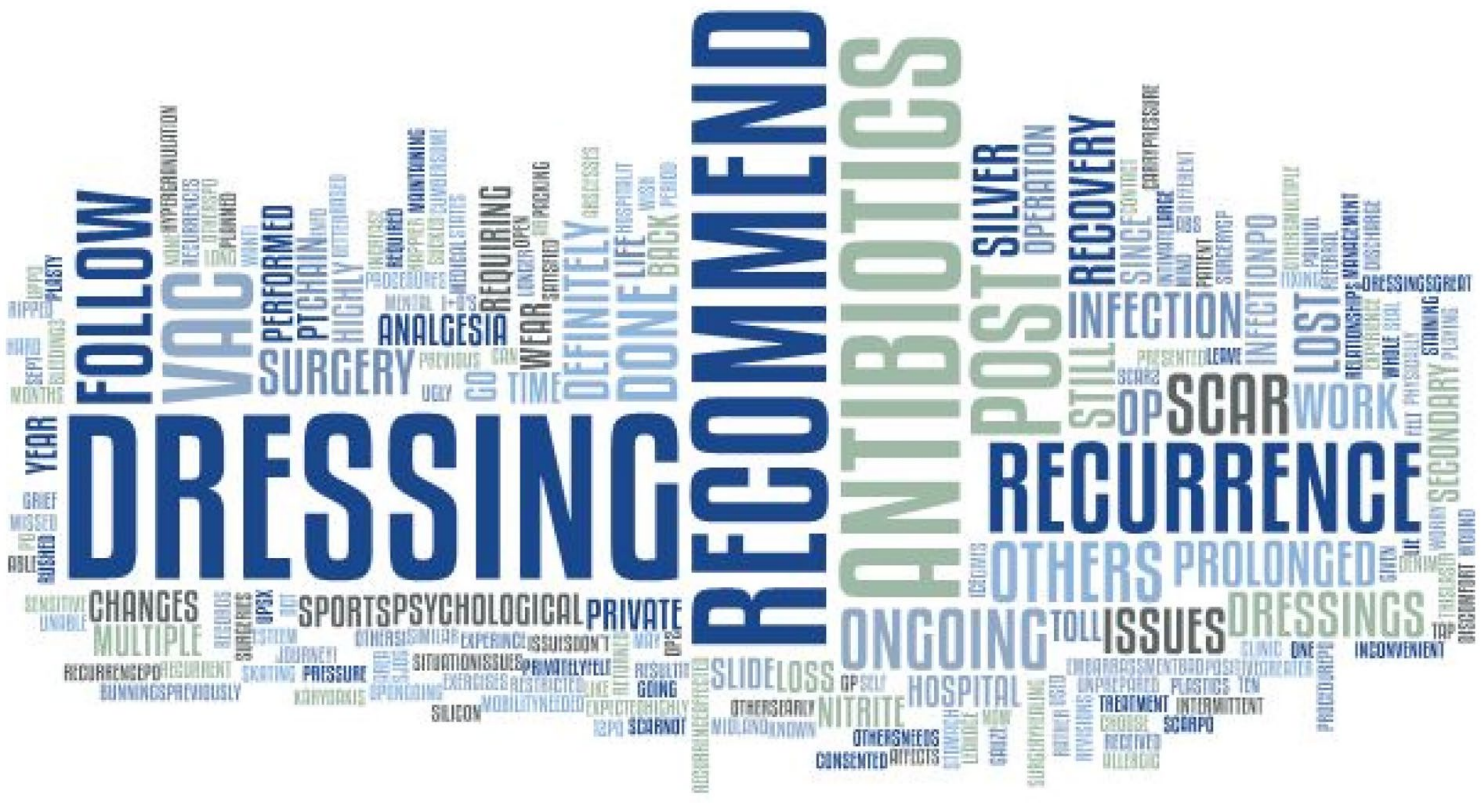
Logarithmic thematic pictorial representation of patient‐reported perspectives and perceptions.

Qualitative responses were mostly positive, though some reported lasting psychological and emotional effects. This was mainly due to embarrassment and reduced self‐esteem, which have a significant impact on social life and intimate relationships and warrant a more in‐depth analysis beyond the scope of this study (Figure 2).

## 4 Discussion

This multi‐centre retrospective and participant follow‐up study offers a detailed Australian review of patient‐reported outcome measures (PROMs) after elective surgery for sacrococcygeal pilonidal sinus disease (SPD). Among six surgical techniques, both clinical and statistical differences were found in functional recovery, cosmetic satisfaction, body image and overall satisfaction. These findings highlight the importance of including PROMs in the assessment of SPD management, alongside traditional clinical outcomes such as recurrence and surgical site infection (SSI) rates.

Techniques such as the modified Limberg flap (MLF) and other flap techniques (OFT) were associated with the fastest functional recovery, allowing earlier return to work and daily routines. In comparison, secondary intention healing (SIT) consistently showed the slowest recovery across all measured domains, with notably longer median times to achieve painless mobilisation and sitting. The difference was particularly striking for return to work, where the median for MLF was only 14 days compared to 60 days for SIT. The extended wound healing trajectory and the need for ongoing wound care inherent to large open defects probably explain the extended recovery observed with SIT.

Body image and cosmetic satisfaction were highest among patients undergoing the modified Karydakis flap (MKF) and Limberg flap (LF), which may be attributable to the symmetrical contour restoration achieved by these techniques [3, 5, 11, 13, 23, 25]. OFT and SIT had the lowest scores in these domains, possibly reflecting less predictable scarring or visible tissue loss. Interestingly, MLF produced a high overall satisfaction score despite not having the highest cosmetic or body image ratings, suggesting that patients may prioritise functional recovery and reduced recurrence risk over purely aesthetic considerations.

Confidence scores declined postoperatively across the cohort, although the clinical significance of this change remains uncertain. The decline was most pronounced in MLF, while LF maintained a stable median confidence level, despite statistical significance, likely due to distributional effects. This highlights the need for further qualitative research into the psychosocial aspects of SPD surgery, particularly regarding preoperative expectations and postoperative support.

Recurrence and complication rates also varied substantially between techniques. SIT demonstrated the highest recurrence rate at 52.2% and the highest SSI rate at 30.4%, which likely contributed to its poorer PROMs and delayed recovery. MLF and MKF achieved lower recurrence rates while maintaining strong PROM performance, offering a favourable balance between functional and aesthetic outcomes despite the greater operative dissection required.

These results align with existing literature that supports off‐midline flap techniques for enhanced cosmetic and body image satisfaction [11, 28, 29]. However, they diverge from traditional assumptions that simpler excisional procedures inherently allow faster recovery, instead showing that well‐executed flap techniques can facilitate earlier mobilisation through improved wound stability and reduced postoperative tension [7, 25, 28]. The poor PROMs associated with SIT are consistent with other reports that highlight prolonged healing, greater wound care burden and reduced patient satisfaction [15, 30].

The strengths of this study include its multicentre design, relatively large sample size, comprehensive PROM assessment and a minimum follow‐up period of 12 months up to 10 years, allowing for the collection of data on both recovery and recurrence. However, the limitations are notable and should be considered when interpreting these findings. The retrospective approach means that procedure choice was influenced by surgeon preference, patient factors and disease severity rather than randomisation, which may introduce selection bias. The study involved three metropolitan services covering eight public hospitals, all situated in Western Australia, potentially limiting the generalisability of the results to other populations and healthcare systems.

PROMs were gathered at different times post‐surgery, which could lead to recall bias despite the use of blinded, standardised questionnaires. The reliance on self‐reported data also means individual perceptions, coping strategies and unrelated health factors could have affected responses. The quasi‐random recruitment method and incomplete response rates raise the possibility of responder bias, with patients experiencing particularly positive or negative outcomes being more likely to respond.

Additionally, several relevant factors, such as exact cleft morphology, disease severity, occupational sitting demands and hair removal practices, were not recorded, restricting the exploration of pathogenesis‐related differences in outcomes. Finally, although the follow‐up duration was sufficient to identify early and intermediate recurrences, pilonidal disease can recur many years after surgery [27]; thus, prospective, longer‐term follow‐up across all participants is necessary to evaluate the durability of both clinical and PROM results fully.

These limitations highlight the need for prospective, preferably randomised, multi‐centre studies using standardised PROM tools and detailed anatomical assessments. Such research would establish a stronger evidence base to support patient‐centred surgical decisions and reduce the uncertainty inherent in retrospective comparisons.

From a practical perspective, these results demonstrate that surgical decision‐making for SPD should be guided by patient priorities, weighing recovery time, recurrence risk and aesthetic results. The modified Limberg and Karydakis flaps seem to provide a favourable balance for many patients. Conversely, healing by secondary intention with negative pressure wound therapy should only be considered when flap closure is not possible, such as in cases of extensive tissue loss or persistent infection, both of which are relative contraindications to reconstruction.

The findings also support the mechanical model of SPD pathogenesis, in which hair entrapment within a broad intergluteal contact surface plays a key role [10]. Flap procedures modify cleft anatomy and decrease the contact surface area, potentially reducing the chance of recurrence by targeting the root cause [15, 16, 28]. This anatomical modification may help explain their superior PROMs. Conversely, SIT leaves the predisposing anatomy unchanged, which likely leads to higher recurrence rates and lower satisfaction.

Nevertheless, translating comparative outcome data directly into patient‐choice models remains a challenging task. It is neither realistic nor helpful for patients to select from a smorgasbord of surgical techniques, especially since most surgeons do not perform all procedures to the same standard. Increasing support exists for the idea that focusing on one well‐performed flap technique, refined through experience and used within a step‐by‐step treatment plan, may produce better overall results than spreading expertise across multiple approaches. This supports calls for broader consensus‐driven treatment algorithms developed with input from both clinicians and patients, aiming to reduce practice variation, improve outcomes and base surgical strategies on the best available evidence.

## 5 Conclusion

This multi‐centre retrospective study identified substantial differences in PROMs across six surgical techniques for SPD. Flap‐based approaches, particularly the modified Limberg and Karydakis flaps, offered the best balance between quick functional recovery, acceptable cosmetic results and lower recurrence rates. Conversely, healing by secondary intention was linked to slower recovery, worse PROMs and increased recurrence.

These findings emphasise the importance of incorporating PROMs into surgical assessment and preoperative counselling for SPD, ensuring clinical decision‐making reflects both surgeon expertise and patient priorities. They also underscore the need for a coordinated approach to surgical practice that reduces unnecessary variation, promotes best‐practice techniques and aligns operative strategies with the underlying pathophysiology of the disease. Future prospective, consensus‐driven research is crucial to develop and implement evidence‐based treatment algorithms that consistently deliver high‐quality outcomes for patients with SPD.

## Conflicts of Interest

The authors declare no conflicts of interest.

## Supporting information


**Data S1:** ans70330‐sup‐0001‐supinfo.docx.

## Data Availability

The datasets analysed in this study are not publicly available due to privacy restrictions. De‐identified data may be obtained from the corresponding author upon reasonable request and with appropriate ethics approval.
