# Supplementary material for: Sacrococcygeal Pilonidal Sinus Disease: A Decade‐In‐Review of Patient‐Reported Outcome Measures
Source: ANZ J Surg. 2025 Oct 11;96(3):525–36. doi: 10.1111/ans.70330 (PMC13006718; doi:10.1111/ans.70330)
Supplement: Supplementary file 1 — Data S1: ans70330‐sup‐0001‐supinfo.docx. [file ANS-96-525-s001.docx]

**Title: Sacrococcygeal Pilonidal Sinus Disease: A Decade-in-Review of Patient-Reported Outcome Measures**

**Supplementary Materials:**

**Authors:**

**1. Dr Munyaradzi G Nyandoro MBBS (Hons), MSurg, BNurs, FRACS,** The University of Western Australia and Fiona Stanley Hospital

**2. Dr Mary MK Teoh MD, BSc (Physio)**, Sir Charles Gairdner Hospital

**3. Dr Ellen G Maclean MD, BSc,** Sir Charles Gairdner Hospital

**4. Dr Andrew Thompson MBBS, FRACS,** Rockingham General Hospital

**5. Professor David Fletcher AM, MBBS, MD, FRACS, GAICD.** Harry Perkins Institute of Medical Research, Fiona Stanley Hospital

Figures, Tables and Captions

| **Supplementary Table 1. (st.1)**Patient Questionnaire | | | | | | | | | | | | | | | | | | | | |  |
| --- | --- | --- | --- | --- | --- | --- | --- | --- | --- | --- | --- | --- | --- | --- | --- | --- | --- | --- | --- | --- | --- |
| After your initial operation did you get a recurrence of your Pilonidal sinus disease? | | | | | | | | | | | | | | | | | | | | |  |
| If YES, how long after your procedure did you get recurrence of Pilonidal disease? (Days) | | | | | | | | | | | | | | | | | | | | |  |
| Did you have a post-surgical wound infection? | | | | | | | | | | | | | | | | | | | | |  |
| If YES, how long after your procedure did you have a post-surgical wound infection? (Days) | | | | | | | | | | | | | | | | | | | | |  |
| Did you need further surgery to manage the recurrence/infection? | | | | | | | | | | | | | | | | | | | | |  |
| In your own estimation, how much time did it take you before: | | | | | | | | | | | | | | | | | | | | |  |
| Painless mobilisation (Days) | | | | | | | | | | | | | | | | | | | | |  |
| Painless sitting on the toilet (Days) | | | | | | | | | | | | | | | | | | | | |  |
| Analgesics no longer required (Days) | | | | | | | | | | | | | | | | | | | | |  |
| Return to usual activities (Days) | | | | | | | | | | | | | | | | | | | | |  |
| Return to work (Days) | | | | | | | | | | | | | | | | | | | | |  |
| Body Image Questionnaire Consisting of a Body Image Score (Items 5 to 9), Cosmetic Score (Items 10 to 12) and Self-confidence (Items 13 to 14) | | | | | | | | | | | | | | | | | | | | |  |
| **BODY IMAGE *(Items 5 to 9), [Best: 20; worst: 5]*** | | | | | | | | | | | | | | | | | | | | |  |
| Are you less satisfied with your body since the operation? | | | | | | | | | | | | | | | | | | | | |  |
| 1. Yes, extremely | | | 2. Quite a but | | | | | | | 3. A little bit | | | | | | | 4. No, not at all | | | |  |
| Do you think the operation has damaged your body? | | | | | | | | | | | | | | | | | | | | |  |
| 1. Yes, extremely | | | 2. Quite a but | | | | | | | 3. A little bit | | | | | | | 4. No, not at all | | | |  |
| Do you feel less attractive as a result of your operation? | | | | | | | | | | | | | | | | | | | | |  |
| 1. Yes, extremely | | | 2. Quite a but | | | | | | | 3. A little bit | | | | | | | 4. No, not at all | | | |  |
| Do you feel less feminine/masculine as a result of your operation? | | | | | | | | | | | | | | | | | | | | |  |
| 1. Yes, extremely | | | 2. Quite a but | | | | | | | 3. A little bit | | | | | | | 4. No, not at all | | | |  |
| Is it difficult to look at yourself naked? | | | | | | | | | | | | | | | | | | | | |  |
| 1. Yes, extremely | | | 2. Quite a but | | | | | | | 3. A little bit | | | | | | | 4. No, not at all | | | |  |
| **COSMETIC SCORE *(Items 10 to 12), [Best: 24; worst: 3]*** | | | | | | | | | | | | | | | | | | | | |  |
| On a scale from 1 to 7, how satisfied are you with your (incisional) scar? | | | | | | | | | | | | | | | | | | | | |  |
| 1. Very unsatisfied | | 2. | | 3. | | | | | 4. Indifferent | | | 5. | | | | 6. | | | 7. Very satisfied | |  |
| On a scale from 1 to 7, how would you describe your (incisional) scar? | | | | | | | | | | | | | | | | | | | | |  |
| 1. Very unsatisfied | | 2. | | 3. | | | | | 4. Indifferent | | | 5. | | | | 6. | | | 7. Very satisfied | |  |
| Could you score your own (incisional) scar on a scale from 1 - 10? | | | | | | | | | | | | | | | | | | | | |  |
| 1. Revolting | | 2 | | | 3 | 4 | | 5. Indifferent | | | 6 | | | 7 | 8 | | | | 9 | 10. Very beautiful | |
| **SELF-CONFIDENCE *(Items 13 to 14), [Best: 10; worst: 1]*** | | | | | | | | | | | | | | | | | | | | |  |
| How confident were you BEFORE your operation, on a scale from 1 - 10? | | | | | | | | | | | | | | | | | | | | |  |
| 1. Not very confident | | 2 | | | 3 | 4 | | 5. Indifferent | | | 6 | | | 7 | 8 | | | | 9 | 10. Very confident | |
| How confident were you AFTER your operation, on a scale from 1 - 10? | | | | | | | | | | | | | | | | | | | | |  |
| 1. Not very confident | | 2 | | | 3 | 4 | | 5. Indifferent | | | 6 | | | 7 | 8 | | | | 9 | 10. Very confident | |
| Overall, how satisfied were you with the outcome of your operation, on a scale from 1 - 10? | | | | | | | | | | | | | | | | | | | | |  |
| 1. Very unsatisfied | | 2 | | | 3 | 4 | | 5. Indifferent | | | 6 | | | 7 | 8 | | | | 9 | 10. Very satisfied | |
| **How strongly do you agree or disagree with each of the following statements?** | | | | | | | | | | | | | | | | | | | | |  |
| At the time of surgery, I feel that I was fully informed about the procedure/risks/benefits involved. | | | | | | | | | | | | | | | | | | | | |  |
| 1. Strongly agree | 2. Agree | | | | | | 3. Neutral | | | | | | 4. Disagree | | | | | 5. Strongly disagree | | |  |
| **At the time of surgery, I feel that I was fully informed about the prognosis of my condition.** | | | | | | | | | | | | | | | | | | | | |  |
| 1. Strongly agree | 2. Agree | | | | | | 3. Neutral | | | | | | 4. Disagree | | | | | 5. Strongly disagree | | |  |
| **At the time of surgery, I feel that I was fully informed about the alternative treatments that were available to me.** | | | | | | | | | | | | | | | | | | | | |  |
| 1. Strongly agree | 2. Agree | | | | | | 3. Neutral | | | | | | 4. Disagree | | | | | 5. Strongly disagree | | |  |
| **I feel that the pre-op clinic consults fully prepared me for the issues/problems I faced post-operatively.** | | | | | | | | | | | | | | | | | | | | |  |
| 1. Strongly agree | 2. Agree | | | | | | 3. Neutral | | | | | | 4. Disagree | | | | | 5. Strongly disagree | | |  |
| **I would have liked to have more time during the pre-op clinics to ask questions and clarify issues.** | | | | | | | | | | | | | | | | | | | | |  |
| 1. Strongly agree | 2. Agree | | | | | | 3. Neutral | | | | | | 4. Disagree | | | | | 5. Strongly disagree | | |  |
| **I fully understand how to manage my disease.** | | | | | | | | | | | | | | | | | | | | |  |
| 1. Strongly agree | 2. Agree | | | | | | 3. Neutral | | | | | | 4. Disagree | | | | | 5. Strongly disagree | | |  |
| Would you recommend the operation to others? | | | | | | | | | | | | | | | | | | | | |  |
| Do you have anything else to add? | | | | | | | | | | | | | | | | | | | | |  |
| ***Standardised patient questionnaire*** | | | | | | | | | | | | | | | | | | | | |  |

| **Supplementary Table 2. (st.2)**Surgical Technique Definitions |
| --- |
| **Karydakis flap (KF)** – Involves an asymmetric elliptical excision of the sinus tract complex with lateralisation of the wound to avoid the midline. A fasciocutaneous flap is mobilised from the contralateral buttock and advanced to cover the defect. This approach flattens the natal cleft, reducing hair accumulation, friction, and maceration, and places the suture line laterally to lower recurrence risk. |
| **Modified Karydakis flap (MKF)** – Builds upon KF principles with modifications including a deliberate tilt that results in further lateralization of the lower wound edge from the midline, whilst incorporating broader subcutaneous undermining, flap thinning to match surrounding tissue, and the use of progressive tension sutures to reduce dead space and wound tension. Closure is performed in multiple layers with absorbable sutures, aiming to optimise contour and healing. |
| **Limberg flap (LF)** – The Limberg flap is a rhomboid transposition flap designed to excise the diseased tissue and flatten the natal cleft. The procedure begins with an excision of the pilonidal sinus in the shape of a rhombus, with each angle measuring 60° and 120°, ensuring complete removal of affected tissue. A flap of adjacent skin and subcutaneous tissue, of identical rhomboid dimensions, is then raised from one side of the defect and transposed to cover the excised area. The donor site is closed primarily. |
| **Modified Limberg flap (MLF)** – As in the standard Limberg flap, the sinus tract and surrounding diseased tissue are excised in a rhomboid configuration. The modification involves altering flap dimensions and orientation to achieve a more lateralised closure, ensuring the midline is only crossed once and the lower edge of the suture line is shifted further away from the midline and with further flattening the natal cleft. In some variations, the flap is extended or rotated to increase coverage and reduce tension, with careful undermining to preserve vascularity. |
| **Other Flap Techniques (OFT)** – This category includes Bascom cleft lift, gluteus maximus myocutaneous rotational flap, Z-plasty, and V-Y advancement procedures. Due to the small number of cases for each individual technique, they were grouped for analysis. Although differing in flap design and tissue handling, all aim to excise diseased tissue, achieve durable defect coverage, and flatten the natal cleft. These approaches are adaptable to variations in anatomy and defect size and are typically selected based on surgeon preference and intraoperative considerations. |
| **Secondary intention technique (SIT)** – Wide excision of the sinus and affected skin without primary closure. The wound is left open to heal via granulation and epithelialisation. This technique avoids suture-related complications but often requires prolonged wound care, delays return to function and may carry a higher recurrence risk. |
| ***Brief description of surgical techniques*** |
